# Collaboration Around Rare Bone Diseases Leads to the Unique Organizational Incentive of the Amsterdam Bone Center

**DOI:** 10.3389/fendo.2020.00481

**Published:** 2020-08-11

**Authors:** Elisabeth M. W. Eekhoff, Dimitra Micha, Tymour Forouzanfar, Teun J. de Vries, J. Coen Netelenbos, Jenneke Klein-Nulend, Jack J. W. A. van Loon, Wouter D. Lubbers, Lothar Schwarte, Patrick Schober, Pieter G. H. M. Raijmakers, Bernd P. Teunissen, Pim de Graaf, Adriaan A. Lammertsma, Maqsood M. Yaqub, Esmée Botman, Sanne Treurniet, Bernard J. Smilde, Arend Bökenkamp, Anco Boonstra, Otto Kamp, Jakko A. Nieuwenhuijzen, Marieke C. Visser, Hans J. C. Baayen, Max Dahele, Guus A. M. Eeckhout, Thadé P. M. Goderie, Cas Smits, Marjolijn Gilijamse, K. Hakki Karagozoglu, Paul van de Valk, Chris Dickhoff, Annette C. Moll, Frank F. D. Verbraak, Katie K. R. Curro-Tafili, Ebba A. E. Ghyczy, Thomas Rustemeyer, Peeroz Saeed, Alessandra Maugeri, Gerard Pals, Angela Ridwan-Pramana, Esther Pekel, Ton Schoenmaker, Willem Lems, Henri A. H. Winters, Matthijs Botman, Georgios F. Giannakópoulos, Peter Koolwijk, Jeroen J. W. M. Janssen, Peter Kloen, Nathalie Bravenboer, Jan Maerten Smit, Marco N. Helder

**Affiliations:** ^1^Amsterdam UMC, Department of Internal Medicine Section Endocrinology, Amsterdam Bone Center, Amsterdam Movement Sciences, Amsterdam, Netherlands; ^2^Amsterdam UMC, Department of Clinical Genetics, Amsterdam Bone Center, Amsterdam Movement Sciences, Amsterdam, Netherlands; ^3^Amsterdam UMC, Department of Oral and MaxilloFacial Surgery/Oral Pathology, Amsterdam Bone Center, Amsterdam Movement Sciences, Amsterdam, Netherlands; ^4^Department of Periodontology, Academic Centre for Dentistry Amsterdam (ACTA), University of Amsterdam and Vrije Universiteit, Amsterdam, Netherlands; ^5^Department of Oral Cell Biology, Academic Centre for Dentistry Amsterdam (ACTA), University of Amsterdam and Vrije Universiteit Amsterdam, Amsterdam Movement Sciences, Amsterdam, Netherlands; ^6^Amsterdam UMC, Department of Anaesthesiology, Amsterdam, Netherlands; ^7^Amsterdam UMC, Department of Radiology and Nuclear Medicine, Amsterdam, Netherlands; ^8^Amsterdam UMC, Emma Children's Hospital, Vrije Universiteit Amsterdam, Department of Pediatric Nephrology, Amsterdam, Netherlands; ^9^Amsterdam UMC, Department of Pulmonology, Amsterdam, Netherlands; ^10^Amsterdam UMC, Department of Cardiology, Amsterdam, Netherlands; ^11^Amsterdam UMC, Department of Urology, Amsterdam, Netherlands; ^12^Amsterdam UMC, Department of Neurology, Amsterdam, Netherlands; ^13^Amsterdam UMC, Department of Neurosurgery, Amsterdam, Netherlands; ^14^Amsterdam UMC, Department of Radiation Oncology, Amsterdam, Netherlands; ^15^Amsterdam UMC, Department Psychiatry, Amsterdam, Netherlands; ^16^Amsterdam UMC, Department of Otolaryngology—Head and Neck Surgery, Ear and Hearing, Amsterdam, Netherlands; ^17^Amsterdam UMC, Department of Otolaryngology—Head and Neck Surgery, Ear and Hearing, Amsterdam Public Health Research Institute, Amsterdam, Netherlands; ^18^Amsterdam UMC, Department of Pathology, Amsterdam, Netherlands; ^19^Amsterdam UMC, Thoracic and Endocrine Surgery, Department of Surgery and Cardiothoracic Surgery, Cancer Center Amsterdam, Amsterdam, Netherlands; ^20^Amsterdam UMC, AMC, Department of Ophtalmology, Amsterdam, Netherlands; ^21^Amsterdam UMC, Department of Dermatology, Amsterdam, Netherlands; ^22^Amsterdam UMC, Department of Ophtalmology, Amsterdam, Netherlands; ^23^Amsterdam UMC, Department of Clinical Genetics, Amsterdam Bone Center, Amsterdam, Netherlands; ^24^Amsterdam UMC, Dentistry and Prosthodontics Department of Oral and MaxilloFacial Surgery/Oral Pathology, Special Dentistry Foundation, Amsterdam, Netherlands; ^25^Amsterdam UMC, Department of Dietetics, Amsterdam, Netherlands; ^26^Amsterdam UMC, Department of Reumatology, Amsterdam, Netherlands; ^27^Amsterdam UMC, Department of Plastic, Reconstructive and Hand Surgery, Amsterdam Bone Center, Amsterdam, Netherlands; ^28^Amsterdam UMC, Department of Trauma Surgery, Amsterdam, Netherlands; ^29^Amsterdam UMC, Department of Physiology, Amsterdam Cardiovascular Science, Amsterdam, Netherlands; ^30^Amsterdam UMC, Department of Hematology, Amsterdam, Netherlands; ^31^Amsterdam UMC, Department of Orthopaedic Surgery, Amsterdam, Netherlands; ^32^Amsterdam UMC, Department of Clinical Chemistry, Amsterdam Bone Center, Amsterdam Movement Sciences, Amsterdam, Netherlands

**Keywords:** rare bone diseases, amsterdam bone center (ABC), collaborative organization, non-hierarchical, research, clinical

## Abstract

In the field of rare bone diseases in particular, a broad care team of specialists embedded in multidisciplinary clinical and research environment is essential to generate new therapeutic solutions and approaches to care. Collaboration among clinical and research departments within a University Medical Center is often difficult to establish, and may be hindered by competition and non-equivalent cooperation inherent in a hierarchical structure. Here we describe the “collaborative organizational model” of the Amsterdam Bone Center (ABC), which emerged from and benefited the rare bone disease team. This team is often confronted with pathologically complex and under-investigated diseases. We describe the benefits of this model that still guarantees the autonomy of each team member, but combines and focuses our collective expertise on a clear shared goal, enabling us to capture synergistic and innovative opportunities for the patient, while avoiding self-interest and possible harmful competition.

## Introduction

Rare bone diseases (RBD) have, until recently, been a largely neglected area in healthcare. Their rarity and heterogeneity have unfortunately hindered their exploration at both clinical and scientific levels, even though more than 500 of the ~7,000 rare diseases are bone disorders (1, 2). The estimated incidence of RBD can vary, from around 15.7/100000 births for skeletal dysplasias (3) which are the most common, to ultra-rare disorders of which only a few patients exist in the world, such as spondylo-ocular syndrome (4). However, in the last decade, the urgency to study and treat RBD has been boosted by the greater appreciation of the socioeconomic consequences associated with their chronic nature and severity, and by the wider availability of genetic diagnostics, patient advocacy, and the development of new pharmaceutical treatment options.

The focus of the Amsterdam UMC initially included the rare bone diseases (RBD) fibrodysplasia ossificans progressiva, osteogenesis imperfecta, fibrous dysplasia and hereditary osteoporosis, but encountered several obstacles. RBD are often extremely challenging to treat; clinical decisions are hindered by their complexity and lack of knowledge about their underlying pathology. Because standard treatment protocols do not exist for RBD, and off-label medications are typically required, a broad team of medical specialists is needed to design the right treatment approach for the individual patients. Ideally, because these diseases are so rare, such a team would be embedded in a multidisciplinary academic setting to facilitate urgently needed clinical and preclinical research. This provides access to research-oriented colleagues who have knowledge and affinity with relevant RBD and increases the likelihood of new insights and scientific breakthroughs to ultimate benefit the patients. Critical to maximizing progress is full collaboration between many different disciplines in a structure, where not only clinicians, but also clinical and basic researchers can efficiently interact across specialities and facilities. Such team structures and broad collaborative networks can be challenging to set up in academic centers due to other interests, competition, or non-equivalent cooperation (5).

### Collaborative Organizational Model

Different opinions exist about the ideal organizational structure to facilitate successful cooperation of professionals from a wide variety of disciplines. Nonetheless, in most medical and research organizations, the traditional hierarchical pyramid still dominates. Such rigidly structured organizations that are managed “top-down” often fail to provide an optimum environment for self-motivation, creativity, engagement, and empathy, all important requirements for effective collaboration amongst colleagues (6–10).

An alternative approach supports a less rigid hierarchy and the promotion of organic development of collaboration between colleagues in a culture of equality (3–7). Fundamental to this is the recognition of the specific and complementary skills of each individual team member. There is increasing support of the idea that teams containing like-minded people with mutual and aligned interests can provide the basis for transparent, fair, and fruitful collaboration. Organizational models like this can achieve shared goals by stimulating an engaged, unforced and valued workforce mentality, in which individuality and freedom to show initiative is safeguarded (6–10). In such a model the aim is not the integration of all departments but an efficient collaboration between relevant partners driven by their balanced skills that are required to solve specific clinical or research questions. The overall goal is to improve patient care and to stimulate innovative research. The process is further enhanced by the critical input of patients in care and research. This kind of model is referred to in the literature as “collaborative organization,” and is considered an effective means of advancing both efficiency and innovation (6–10).

## Amsterdam Bone Center

The Amsterdam Bone Center (ABC) was formed in late 2016, as a successful example of such “collaborative organizational model.” The ABC was an initiative of various clinical disciplines and researchers who wanted to pool their specialized skills, knowledge and experience across boundaries and their day to day scope, with the common goal of achieving new approaches to the diagnosis, care, and effective treatment of patients with RBD. Most of RBD treatment is still based on generic medical protocols which provide symptomatic relief, but effective future therapies that result in the recovery of the affected tissue will need detailed understanding of the underlying disease pathology, which is a challenging task. As a consequence, the ABC was initially focussed on RBD. Although the number of patients affected by some diseases was very limited, the level of required adapted complex care was very high. This resulted in extensive networking with many clinical departments such as plastic surgery, maxillofacial surgery, orthopedic surgery, thoracic surgery, traumatology, anaesthesiology, rehabilitation, urology, ear nose and throat surgery, audiology, ophthalmology, clinical genetics, rehabilitation, psychiatry, physiotherapy, social work, dietetics, gypsum master, cardiology, lung disease, nuclear medicine, radiology, neurology, neurosurgery, dermatology, radiotherapy, gastroenterology, endocrinology, pediatrics, rheumatology, and dentistry. In addition, the patient organizations have been actively involved. The multidisciplinary collaboration has been based on equality.

The ABC subsequently developed as a flat organization, where mutual interest, exchange of knowledge, and innovation have led to a vivid open collaboration between clinicians and researchers.

The ABC provides a bridge between clinicians and research laboratories whose partners are embedded in the Amsterdam Movement Sciences research institute, the latter of which embraces the targeted laboratories specializing in multifaceted aspects of research on bone tissue, dentition and the surrounding tissues. In this way, it connects expert groups focussed on osteocytes (11), osteoblasts (12), osteoclasts (13), bone matrix formation (14), and angiogenesis (15), facilitating the study of bone differentiation and regeneration. With the aid of appropriate cell collection from RBD and control tissues, complex processes can be studied and interpreted in the physiological and pathological context. “Meet the expert” RBD sessions and annual RBD meetings help to keep the patients informed about the current research and progress. ABC education activities also extend to academic training at the bachelor, master and doctorate level by which enthusiasm for rare bone diseases is promoted in talented young professionals.

## Management of the ABC

In place of the more typical hierarchical model in which all control is centralized to a Director, the ABC operates with a facilitating steering team, with one member in rotation functioning as the ABC chairman. The chairman conveys the consensus goals, ambitions, and decisions of the team. The different cultures and perspectives of the various collaborating departments are reflected in a steering team of four coordinators from the task force group, consisting of 2 preclinical theme leaders (from the Laboratory for Bone Metabolism of the Department of Clinical Chemistry and Cell Technology Laboratory of the Department of Oral and Maxillofacial Surgery) and 2 clinical theme leaders (from the Department of Internal Medicine section Endocrinology and the Department of Plastic Surgery). Steering team members are elected to their role for 2 years, based on their proven commitment to the ABC and their activities in promoting its interests.

**Figure 1:**
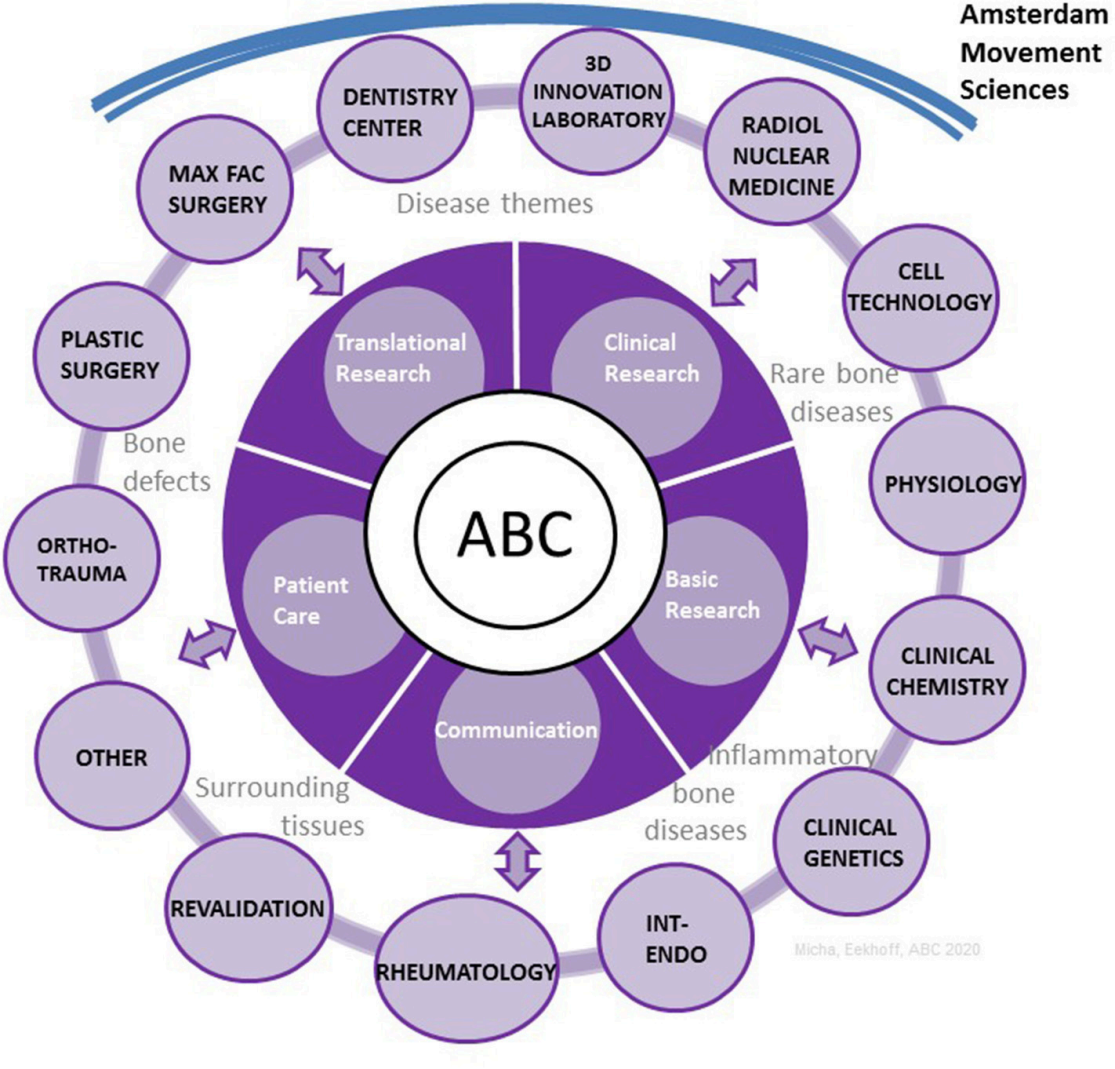
The organizational structure of the ABC with the main themes and partnerships. ABC, Amsterdam Bone Center; Max Fac, maxillo facial; Ortho-trauma, orthopedic surgery, traumatology; Int-endo, internal medicine, endocrinology; Radiol, radiology.

In addition to the leading steering team, there is a task force which includes representatives from clinical and pre-clinical groups. These representatives are responsible for promoting their key themes [e.g., key themes are presently RBD, inflammatory bone diseases and bone oncology, complex fractures, and complex surrounding tissue injuries (Figure 1)]. The task force comes together in brainstorm sessions to translate critical clinical questions into structured preclinical research lines, and move preclinical findings into the clinical environment. In this organizational model, the coordinating task force is not focused on safeguarding its own structure, but on leveraging its diverse expertise to drive adoption of new ideas across the ABC members, identify scientific gaps, support the finding of solutions, enhance ABC connectivity and crosstalk between themes where possible, give direction to future common goals, support optimal clinical care for patients, and provide high quality education and research.

An annual symposium will ensure that all groups working in bone research and clinics in the ABC can benefit and easily collaborate in an ideal setup for research and care. Yearly goals are suggested and proposed to the ABC community in these symposia, and subsequently set and evaluated by the task force based on extensive feedback. The ultimate goal is to become further embedded in a larger international network of centers for bone research in general, and RBD in particular, in order to meaningfully help patients and offer innovative diagnostics, to develop treatment options and recovery solutions for RBD and related bone diseases. The task force also monitors whether the activities of the leading steering team align with the goals of the wider ABC community. The obvious advantage of this lateral (“flat”) organization is that groups retain the freedom to pursue their own research choices, but they are encouraged to reach the best joint benefit.

## Focus of RBD Within the ABC

**Figure 2:**
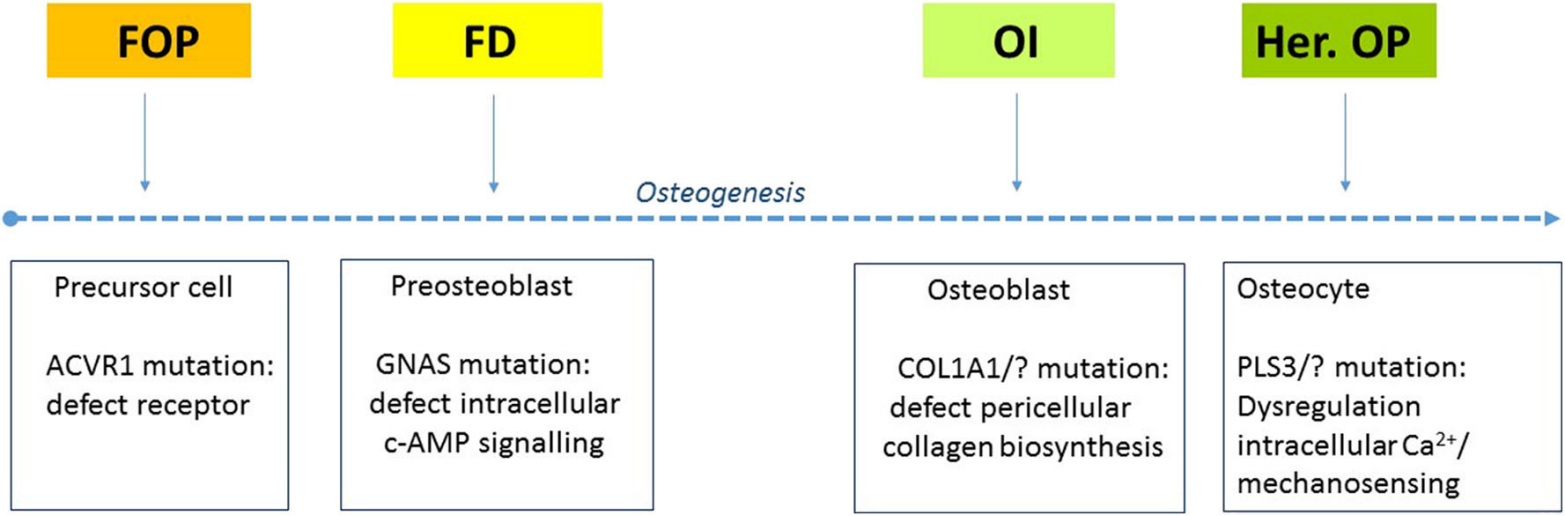
Four genetic rare bone diseases that all dysregulate osteogenesis but in a different phase and on a different process in the cell. The arrow indicates the direction of the stem cell/fibroblast toward the development of osteocytes. FOP, fibrodysplasia ossificans progressiva; FD, fibrous dysplasia; OI, osteogenesis imperfecta; Her. OP, hereditary osteoporosis; ACVR1, activin A receptor 1; c-AMP, Cyclic adenosine monophosphate; COL, collagen; PLS, plastin; Ca, calcium.

The focus of the RBD theme of ABC was initially placed on four RBD, including fibrodysplasia ossificans progressiva (FOP) (12, 13, 16–19), osteogenesis imperfecta (OI) (20, 21), fibrous dysplasia (FD) with an emphasis on skull (22), and hereditary osteoporosis (her. OP) (23). This, repertoire was strategically composed based on the various clinical and research expertise available and on the possibility to match underlying etiology and clinical questions. A schematic overview of the differences and common ground of these RBD is given in Figure 2. Based on this, it is clear that these diseases can serve as a paradigm for other RBD sharing a similar pathology, but also provide insight into general bone pathology.

## Achievements on RBD Within the ABC

**Figure 3:**
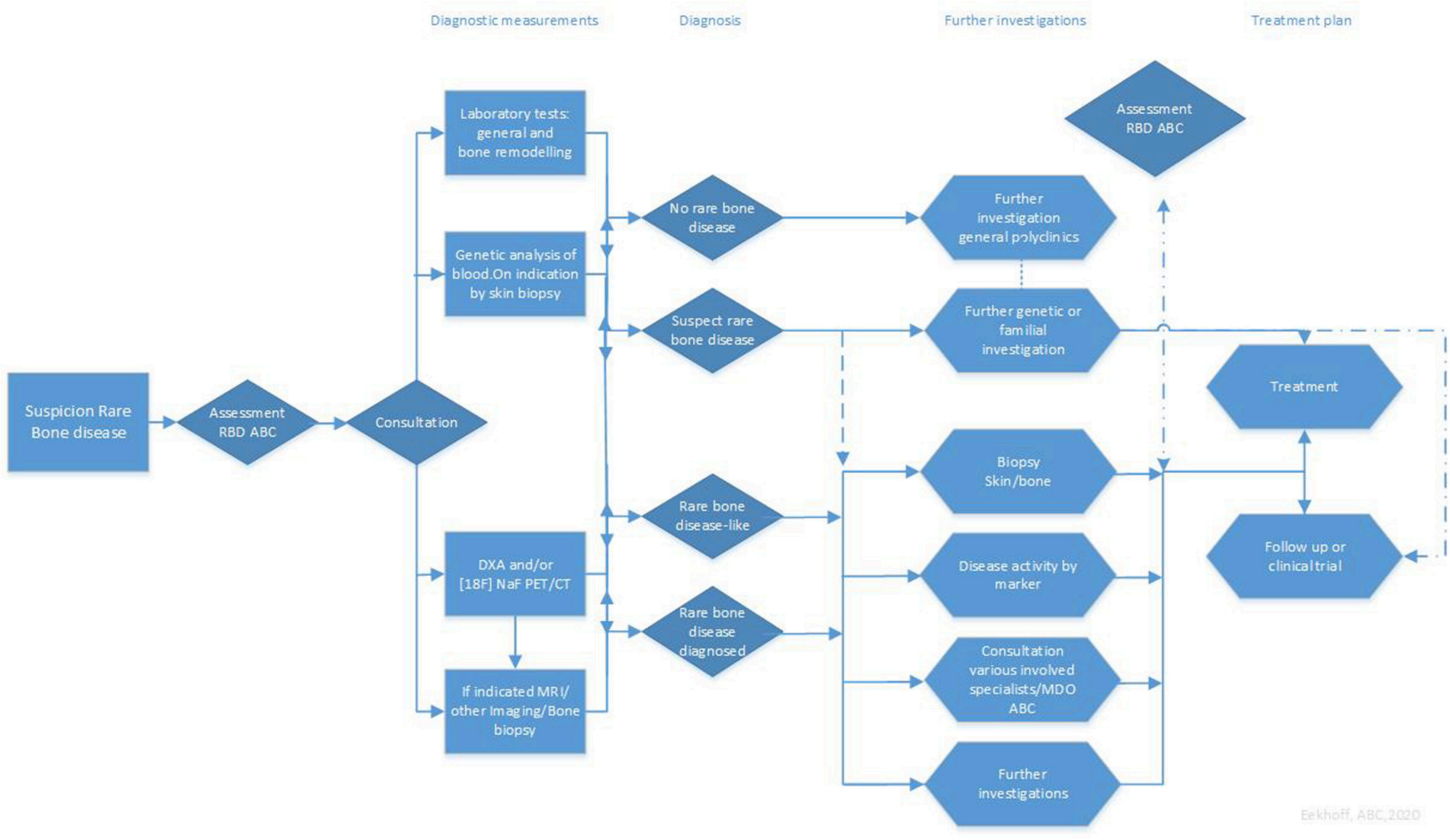
Patient Care Rare Bone Disease ABC, Amsterdam UMC. FOP, fibrodysplasia ossificans progressiva; FD, fibrous dysplasia; OI, osteogenesis imperfecta; Her-OP, hereditary osteoporosis; ABC, Amsterdam bone Center; DXA, Dual X-ray absorptiometry; [18F] NaF PET/CT, ^18^F-Sodium Fluoride positron emission tomograph/-computed tomography; MRI, Magnetic resonance imaging; MDO, multidisciplinary consultation.

A standardized approach to patient care of the four RBD is developed with the relevant clinical disciplines and patient organizations to create a patient-centered design. This has led to the implementation of a standardized route for care; its integration in numerous specialities is designed to thoroughly address all pathological aspects of each RBD (Figure 3). As a spin-off of the ABC structure, the RBD team has become an international referral center for FOP and it coordinates international studies on FOP, OI and hereditary OP, and FD.

Several preclinical research models have been developed to study the various RBD, including the culture of subdermal (12) and periodontal ligament fibroblasts (13) which can be converted to cartilage and bone-forming cells, or can be drivers for osteoclast formation (13, 24); this provides unique insight in rare bone diseases that primarily focuses on additional bone formation rather than affected bone degradation. Many signaling pathways for cartilage and osteogenic differentiation are reflected in these models, which facilitates their study in easily obtainable patient tissue. This collaboration has yielded the discovery of newly discovered genes for these RBD (20, 23); the investigation of their mechanism can help to shed light in possible therapeutic implications. The collaborative efforts have also led to innovative diagnostics, one example of which is a new modality for imaging of active heterotopic bone lesions in FOP patients with ^18^F PET/CT (16–19). Other advances include the development of new clinical trials with existing and new medications, translational projects on pharmacological therapy for RBD, and the development of new technology to quantify osteoclast activity *ex vivo*. The development of the RBD theme within the ABC structure has led to an increasing number of pre- and clinical scholarships, awarded from Amsterdam UMC AMS as well as other international universities, patient associations, and national and European funding organizations, in collaboration with pharmaceutical/industrial companies. This supports a rapidly developing academic trajectory resulting in many Ph.D. projects and dissertations.

## Future Plans of the ABC

Regarding the future treatment of RBD, Regenerative Medicine (RM) is one of the main research priorities of the ABC. This specific focus within ABC aims meaningful repair/regeneration by exploiting the plasticity of the body's own cells. This requires extensive knowledge of the pathological mechanism of the disease extending from molecular interactions at the cellular level, to the influence of and inter-relationship with the surrounding tissues and other systemic factors. The aforementioned preclinical RBD models and findings can potentially be integrated with RM strategies in order to achieve synergy in disease control and tissue regeneration. This specific expertise within ABC extends to more prevalent bone disorders which are genetically less well-defined but which nonetheless may also benefit from therapeutic developments on RBD; these may include multifactorial osteoporosis, and immune-related bone diseases. The ultimate goal is to establish a regeneration center based on the development of new pathophysiological models for the realization of individualized treatment and prevention. In addition, ongoing future plans include the development of orthoplastic centers and the expansion of our network to more national, European and international collaborators outside the Amsterdam UMC.

In conclusion, this article we have outlined the establishment and development of the Amsterdam Bone Center, where “collaborative organization” encourages the cooperation of all relevant clinic and research teams. Specifically, we have successfully established a patient-centered, multidisciplinary focus on RBD including the development of targeted innovative diagnostics, clinical and research protocols and studies. Recognition of the different cultures and perspectives of the departments represented in the ABC, shared collaborative leadership, and a diverse and well-functioning task force is critical to maintaining a balanced and successful collaboration that advances science and innovation, and improves patient care.

Knowledge of this model may be useful to other organizations aiming to establish or enhance the growth of clinical-academic collaboration.

## Data Availability Statement

All datasets presented in this study are included in the article/supplementary material.

## Author Contributions

This article on a unique collaborative, interdisciplinary concept in medical research arising from the rare bone diseases pillar of the Amsterdam Bone Centre was initiated by EE with contributions to all crude versions from DM, TF, TV, JCN, JK-N, JL, PKl, NB, JS, and MH. The subfinal version was prepared and sanctioned by this core group of authors. All remaining authors WDL, LS, PS, PR, BT, PG, AL, MY, EB, ST, BS, ABö, ABoo, OK, JAN, MV, HB, MD, GE, TG, CS, MG, KK, PV, CD, ACM, FV, KC-T, EG, TR, PS, AM, GP, AR-P, EP, TS, WL, HW, MB, GG, PKo, and JJ are active ABC members and have contributed for many years on rare bone diseases and to the success of this unique collaborative initiative. All these authors have had the opportunity to further improve the manuscript, these comments were incorporated by EE and DM in the submitted version.

## Conflict of Interest

The authors declare that the research was conducted in the absence of any commercial or financial relationships that could be construed as a potential conflict of interest. The handling Editor declared a past co-authorship with one of the authors WL.
